# Portal Vein Thrombosis in Patients Without Cirrhosis: Current Practical Approaches and Treatment Strategies

**DOI:** 10.3390/diagnostics15060721

**Published:** 2025-03-13

**Authors:** Fernando Gil-Lopez, Fausto Alfredo Rios-Olais, Lydia A. Mercado, Denise M. Harnois

**Affiliations:** 1 Department of Liver Transplant, Mayo Clinic Florida, Jacksonville, FL 32224, USA; gil-lopez.fernando@mayo.edu (F.G.-L.); mercado.lydia@mayo.edu (L.A.M.); 2 Hematology and Oncology Department, Instituto Nacional de Ciencias Médicas y Nutrición Salvador Zubirán, Tlalpan, Mexico City C.P. 14080, Mexico; fausto.rioso@incmnsz.mx; 3 Department of Gastroenterology and Hepatology, Mayo Clinic Florida, Jacksonville, FL 32224, USA

**Keywords:** non-cirrhotic, portal vein thrombosis, variceal bleeding, thrombophilia, etiology, complications, anticoagulation

## Abstract

Portal vein thrombosis in non-cirrhotic individuals, although uncommon, is an increasingly explored condition that affects mainly young people, consequently representing a significant disease burden. Reports primarily including western European populations have recently shed light regarding the pathophysiology, risk factors, natural history, treatment, and prognosis of this entity. Underlying predisposing conditions are documented in ~70% of cases, encompassing local risk factors, inherited and acquired thrombophilia, cancer, and systemic inflammatory conditions. Non-cirrhotic portal vein thrombosis can cause significant portal hypertension in the acute setting, but, more frequently, significant portal hypertension-related complications arise when the condition becomes chronic and portosystemic collaterals develop, increasing the risk for variceal bleeding and ascites. The diagnostic approach to screen for underlying thrombophilia remains a challenge, and recommendations in this regard, although scarce and backed by scarce evidence, have changed notably in the last years, leaning toward a universal screen in patients who develop this condition without a clear provoking factor. Recently, studies have shown that long-term anticoagulation may be appropriate even in the absence of clear provoking factors or underlying thrombophilia. Future studies should address which patients may benefit from this approach, which patients may not need it, and what the most appropriate strategies are to approach patients who do not recover portal vein patency with anticoagulation to further prevent portal hypertension-related complications.

## 1. Introduction

Portal vein thrombosis (PVT) is defined by the presence of a thrombus in the portal vein trunk with or without obstruction of the rest of the portal venous system, outside the setting of malignancy [1,2]. Most commonly it affects individuals with cirrhosis but can also occur in persons without cirrhosis, where it is usually associated with local or systemic prothrombotic conditions (which will be referred to as “thrombophilia” in the present article) [3].

Outside the setting of cirrhosis, it is a rare disorder, and epidemiological data on its prevalence and incidence are very scarce and limited due to heterogeneity of the described populations. Considering data from western European countries and including PVT in the context of cirrhosis as well, the incidence of PVT is estimated to be ~2 cases per 100,000 inhabitants per year [4,5].

This review presents a concise review for clinicians about the most relevant current concepts in non-cirrhotic non-tumoral PVT in adults. Isolated thromboses of the splenic or mesenteric vein (MV) are beyond the scope of this article. Of note, a substantial amount of the available literature to date regarding this rare entity comes from research studies encompassing western European populations, and refers to or includes patients with cirrhosis, given the notably higher prevalence of PVT in that setting. When appropriate, the inclusion of cirrhotic patients within the cited studies will be clearly specified.

## 2. Classification

**Figure 1:**
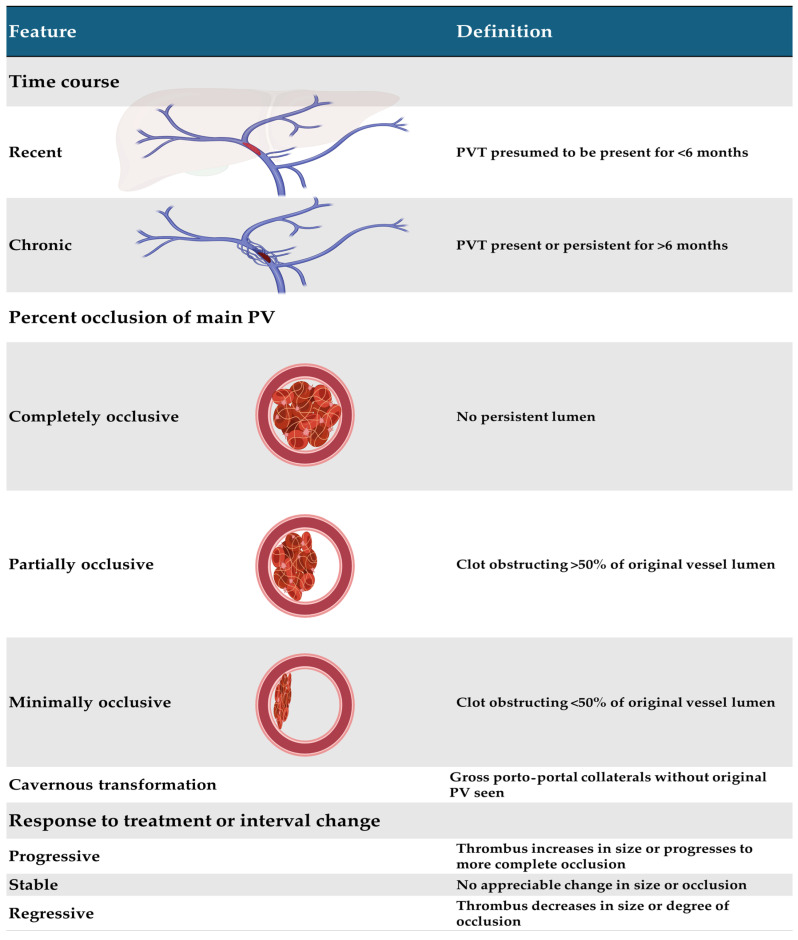
Current proposed classification by AASLD based on time course, percentage of occlusion of the main portal vein, and response to treatment or interval change. Created with BioRender.com using information from Vascular Liver Disorders, Portal Vein Thrombosis, and Procedural Bleeding in Patients with Liver Disease: 2020 Practice Guidance by the American Association for the Study of Liver Diseases [6].

Given the multiple and heterogeneous classifications previously used to designate non-cirrhotic PVT, in an effort to unify the terminology used in clinical and research settings, the American Association for the Study of Liver Diseases (AASLD) proposed a classification in 2021 based on time course, percentage of occlusion of the main portal vein, and response to treatment or interval change (Figure 1). Notably, regarding the time course of PVT, they emphasize the importance of using the term “recent” instead of “acute”, considering that the former implies the presence of symptoms as well as a condition with a recent onset, but some patients do not develop symptoms at all, thus making it difficult to establish precise dating. Therefore, they define ≤6 months as the cut-off point to classify PVT as recent, while it is considered chronic PVT if it is present for more than 6 months [6]. We will use acute and recent interchangeably for clarity purposes when considered appropriate. Interestingly, the proposed 6-month cut-off point is mainly based on the largest prospective PVT longitudinal study in this regard, published by Plessier et al. in 2010 [7], which included 102 patients with “acute” PVT unrelated to cirrhosis who were followed up for 12 months. When recanalization occurred, it happened within the first 6 months after the diagnosis in the vast majority of patients, and cavernous transformation developed in most of those who did not have recanalization [7]. Although multiple studies have proposed classification systems, they were mainly developed including patients with cirrhosis and/or liver transplants to correlate with relevant clinical outcomes in this population. Thus, its usefulness outside that setting is limited [8,9,10].

In summary, besides the mentioned criteria, other important features that should be documented at diagnosis include the extension of the thrombosis, degree of luminal extension, and chronicity of the clot, which is intended to establish a baseline available for comparison during follow-up, particularly for patients who will be treated and whose response to it will require assessment with subsequent imaging [6,11,12].

## 3. Etiology and Risk Factors

Outside the setting of cirrhosis, classic risk factors for PVT include myeloproliferative neoplasm (MPN), abdominal surgery, solid malignancy (mainly hepatobiliary cancer), intra-abdominal inflammatory conditions (diverticulitis, appendicitis, inflammatory bowel disease [IBD], pancreatitis, etc.), and systemic thrombophilia. The presence of more than one risk factor has been reported in 15–36% of patients [13], and in 20–40% no predisposing and/or etiological factor for PVT is identified after a thorough investigation [4,7,13,14].

Among classically reported risk factors, MPN accounts for 41%, antiphospholipid syndrome (APS) for 6–10%, paroxysmal nocturnal hemoglobinuria (PNH) for 1%, factor V Leiden for 3–8%, Factor II G20210A mutation for 3–6%, protein C deficiency for 1–6%, protein S deficiency for 1–5%, antithrombin III deficiency for 1%, pregnancy for 2%, oral contraceptive use for 14–22%, systemic disease (autoimmune diseases, IBD, celiac disease, sarcoidosis, human immunodeficiency virus infection) for 3%, intra-abdominal inflammatory conditions (pancreatitis, intra-abdominal infection) for 11%, abdominal trauma for 4%, and more than one risk factor is present in 14–19% [6,7,13,15,16,17]. It is advised that these numbers be interpreted with caution, given that there are many nuances regarding the appropriate laboratory measurement for some of those parameters, many of which require specialized resources [18] that are not readily available outside of developed countries.

MPNs have long been linked to an increased risk of splanchnic venous thrombosis, and they are consistently the most common identifiable risk factor in patients with non-cirrhotic PVT (21–31%). Within this group, a JAK2 V617F mutation is by far the most common (>80%) [19]. The main mechanisms underlying the increased thrombotic risk of these patients are increased platelet–leukocyte aggregates, increased expression of tissue factor and P-selectin by platelets, and increased levels of thromboxane A2, CD40 ligand, and soluble P-selectin. An elevated hematocrit, along with increased endothelial adherence of mutated red blood cells and enhanced expression of P-selectin and von Willebrand factor by mutated endothelial cells, also promotes leukocyte adhesion and thrombus formation [20].

Malignancy, especially in hepatobiliary cancer, is another well-described risk factor for the development of non-cirrhotic PVT [21]. Cancer cells can activate the coagulation cascade through tissue factor (TF) expression and inflammatory cytokines (TNF-α and IL-1β), consequently stimulating vascular cells, monocytes, and macrophages to express TF, which in turn induces the conversion of factor VII to factor VIIa, thus activating the extrinsic pathway of the coagulation cascade [22,23]. Furthermore, cancer procoagulant, a cysteine protease, stimulates the conversion of factor X to factor Xa [24]. Given the well-defined high thrombotic risk associated with gastrointestinal cancer [25], although it sounds reasonable to screen for malignancy with upper endoscopy and colonoscopy in a patient with newly diagnosed PVT [26], there is no solid evidence in this regard, and thus no formal recommendations from international consensus guidelines, but some authors have recommended not performing an extensive solid malignancy screening in this population, with the argument that imaging studies performed for the diagnosis of PVT are generally sufficient to demonstrate regional occult cancer [27].

Systemic congenital and acquired thrombophilia, such as Factor V Leiden, antithrombin III deficiency, and factor II G20210A mutation, as well as PNH should be considered within the differential diagnosis; splanchnic thrombosis has been reported to present in 7% of PNH cases in a recently published large cohort [15]. Although these disorders are very rare and data are limited, they are considered to be more common in patients with non-cirrhotic PVT compared to the general population, and therefore are recommended as part of the universal diagnostic panel [13,16].

Protein C and protein S deficiency prevalence studies in patients with PVT have yielded equivocal results given that most studies have included patients with chronic liver disease, and the levels of these natural anticoagulants are affected by anticoagulant therapy and acute thrombosis [6,28]. Nevertheless, there are properly defined cut-off values for reference to be considered when assessing for deficiency, provided that the patient has normal baseline coagulation factor levels. There are multiple different assays and considerations (heterozygous or homozygous mutation, laboratory assay, timing of measurement after thrombotic event) that should be considered prior to establishing a diagnosis of true deficiency, which merits consultation with a thrombosis expert [18].

APS has been reported to be an underlying thrombophilia in 6% of patients with PVT, and, reciprocally, PVT has been reported to be a very rare thrombotic manifestation of APS in large European cohorts (~1%) [29,30]. Although data are very scarce in this regard for proper conclusions to be drawn, mixed and unclear results have raised reservations about the utility of testing for antiphospholipid antibodies in this population [31]. However, it is generally recommended to include APS within the work-up of a patient with non-cirrhotic PVT [6,13].

Recently, other less common risk factors such as obesity, SARS-CoV-2 infection or vaccination, and Cytomegalovirus (CMV) infection have gained importance as potential contributors for the development of PVT.

Central obesity, a key component of metabolic syndrome, has been independently linked to an increased risk of non-cirrhotic portal vein thrombosis (PVT). A French study by Bureau et al. found that central obesity, indicated by increased waist circumference and body mass index (BMI), was significantly more common in patients with idiopathic PVT compared to those with secondary PVT and controls from the general population. The study included 79 patients with non-cirrhotic PVT that were divided into two groups: those with secondary PVT (SPVT) and those with idiopathic PVT (IPVT). Individuals who were considered overweight comprised 82% of the IPVT group, compared to 44% of individuals in the SPVT group (*p* = 0.002) and 51% of individuals in the control group (*p* = 0.01). The mean visceral fat area was higher in the IPVT group compared to the SPVT group (18,223 mm^2^ vs. 12,690 mm^2^, *p* = 0.02). An increase in waist circumference was identified as the strongest parameter associated with idiopathic PVT [32]. Given the limitations of the study, the authors acknowledge that it is still unknown if among patients who are overweight, only the ones with metabolic dysfunction-associated fatty liver disease (MAFLD) are at risk for PVT. Another study reported that plasminogen activator 1 promoter polymorphisms (PAI-1) was the only coagulation factor increased in patients with obesity and MAFLD, with a direct correlation with MAFLD severity [33]. A synergistic effect of increased PAI-1, platelet dysfunction, and slower portal flow due to portal hypertension occurring in the context of the hypercoagulable and hypofibrinolytic state of patients with non-cirrhotic MAFLD has been proposed as the mechanism underlying a potential increased risk for developing PVT in this group [34].

SARS-CoV-2 infection has been linked to an increased risk of developing PVT as well. A study published in 2023 reported 21 cases of PVT in the context of COVID-19. SARS-CoV-2 was the only prothrombotic condition encountered in the majority of patients, whereas another concurrent prothrombotic condition was identified in 44% of patients. Given the more severe nature of PVT in patients with confirmed SARS-CoV-2 infection, intestinal infarction leading to intestinal resection was more common (11%) compared to controls (2.6%) without SARS-CoV-2 (*p* = 0.044) [35].

Regarding COVID-19 vaccine-associated PVT, a multicenter study reported 24 patients diagnosed with PVT within 11 weeks after vaccination. Compared to patients with splanchnic vein thrombosis before the COVID-19 pandemic, those with underlying prothrombotic conditions were significantly less common (28% vs. 52%, *p* = 0.01), more frequently required bowel resection for severe mesenteric ischemia (17% vs. 3%, *p* < 0.001), and had significantly worse 1-year mortality (3% vs. 0.2%, *p* = 0.01) [36].

CMV disease (defined as CMV infection with signs or symptoms of disease) was recently established as a solid risk factor for PVT in a multicenter French study, where the authors reported the coexistence of another risk factor for non-cirrhotic PVT in >50% of this population, and 22% of the individuals had a prothrombin G20210A gene variant. Thus, considering the high general population prevalence of CMV infection and the rarity of the mentioned mutation, the authors concluded that CMV disease is not a strong risk factor for PVT, but rather a trigger for PVT in susceptible patients [37].

Porto-sinusoidal vascular disorder (PSVD) is a relatively new entity whose etiology and natural history are not well understood, but it appears to have a strong link with prothrombotic states. PVT is relatively common in patients with PSVD (13–46%), but it remains unclear whether PVT embodies a complication of PSVD, is an etiological factor in its pathogenesis (prothrombotic state, decreased portal flow velocity), or a combination of both. The pathogenesis of this condition is multifactorial, involving a complex interplay of altered intestinal permeability, immune dysregulation, and procoagulant states, leading to endothelial dysfunction and portal hypertension. Increased intestinal permeability leading to translocation of gut-derived endotoxins into the portal circulation, activation of the Toll-like receptor pathway in hepatic macrophages, promotion of inflammation within the portal tracts and sinusoids, mutations in genes such as FCHSD1 (involved in mTOR pathway) leading to aberrant vascular remodeling and endothelial dysfunction, and elevated levels of Factor VII, von Willebrand Factor, and other markers of platelet aggregations and hypercoagulability contributing to microvascular injury and portal hypertension have been implicated in the pathogenesis [38,39]. Autoimmune mechanisms have also been reported, with a higher prevalence of autoantibodies, including anti-endothelial cell antibodies, although with only moderate diagnostic capacity (positive predictive value of 63%) according to a Spanish study that included 37 patients with PSVD and 39 with cirrhosis matched by gender, signs of portal hypertension, and liver function [40]. Finally, exposure to certain drugs such as oxaliplatin can potentially cause sinusoidal endothelial damage through oxidative stress and endothelial cell injury, resulting in sinusoidal obstruction and portal hypertension [41].

The diagnosis of PSVD requires a biopsy, which would demonstrate lesions in the portal veins and sinusoids in the absence of cirrhosis, which includes obliterative portal venopathy, nodular regenerative hyperplasia, and incomplete septal fibrosis in the context of portal hypertension. Imaging studies, including CT scans or MRIs, typically demonstrate intrahepatic abnormalities, focal nodular hyperplasia-like lesions, and abnormal liver morphology, such as peripheral parenchymal atrophy and compensatory hypertrophy of central segments [6,42,43]. On contrast-enhanced MRI, periportal hyperintensity on HBP was recently identified as a very specific radiological feature of PSVD (14 of 33 patients in a European cohort, hypothesized to be due to regenerative changes in periportal hepatocytes leading to a relatively increased enhancement compared to the damaged background) [44]. Finally, the diagnosis also requires the exclusion of other liver diseases related to hepatic vein obstruction and specific causes for microvascular disease (e.g., sarcoidosis, congenital hepatic fibrosis, and schistosomiasis), as well as sinusoidal obstruction syndrome, as defined after hemopoietic stem-cell transplantation and diagnosed according to the Seattle or Baltimore criteria, which should not be considered a form of PSVD [45].

Although in previous years they were considered to be mutually exclusive, it is now accepted that PVT can occur as a complication of PSVD. A retrospective study reported the development of PVT in 30% of patients with PSVD after a median follow-up of 96 months (interquartile range 52, 139). A more recent multicenter prospective study provided significant insights into the natural history and long-term outcomes of porto-sinusoidal vascular disease (PSVD) with portal hypertension (PH). The study included a large multicenter cohort of 587 patients with PSVD, with a median follow-up of 68 months. The 5-year cumulative incidence of developing PVT in patients with PSVD was 16%, signifying a relatively common complication in the natural history of PSVD. The presence of PVT was associated with worse clinical outcomes, including higher rates of hepatic decompensation, exemplified by increased incidences of ascites, variceal bleeding, and hepatic encephalopathy. During the follow-up period, 19% of patients died, and 8.5% underwent liver transplantation.

Thus, it has been proposed that in patients with PVT, PSVD should be actively investigated [46], especially in those without an identifiable thrombophilia, but there are no formal universal guidelines suggesting this approach; therefore, the diagnostic work-up should be considered on a case-by-case basis.

Management strategies to prevent PVT in patients with PSVD are currently being investigated. A French randomized clinical trial including 166 patients (Apixaban for Intrahepatic Non-Cirrhotic Portal Hypertension study [APIS]; ClinicalTrials.gov ID NCT04007289) is currently ongoing to evaluate the efficacy of apixaban in this population to prevent the occurrence or extension of portal, splenic, or mesenteric vein thromboses, and thus the development of chronic portal vein thrombosis and associated complications.

## 4. Diagnostic Approach

It is important to consider that thrombophilia testing has many nuances and controversies given the scarcity of evidence in this regard. It should also be acknowledged that even though great progress has been made in the identification of heritable coagulation defects in a significant proportion of patients with venous thromboembolism, according to some thrombosis experts, testing has not, in general, proven to be useful in the management of this condition [18]. This statement might be debated, but for the purpose of non-cirrhotic PVT, it essentially lies on the established premise that an event of unprovoked splanchnic venous thrombosis (classified as an unusual site venous thrombotic event) would be an indication for long-term anticoagulation, regardless of the presence of a persistent risk factor [27].

The American Society of Hematology (ASH) 2023 guidelines suggest thrombophilia testing to guide anticoagulant treatment duration after primary treatment (6 months) [47] in patients with splanchnic venous thrombosis (SVT) in a situation where anticoagulation would be discontinued; they suggest continuing anticoagulation indefinitely in patients with thrombophilia [48]. For patients with SVT where anticoagulation would be continued indefinitely, they suggest not performing thrombophilia testing. The mentioned suggestions acknowledge that they are conditional recommendations, based on very low certainty in the evidence to support it [48].

On the other hand, the AASLD and the Baveno VII consensus recommend evaluation of patients who develop unprovoked non-cirrhotic PVT by a hematologist and a hepatologist, specifying that the identification of a risk factor does not preclude the need for a complete work-up [1,6], and, thus, a comprehensive inherited and acquired thrombophilia work-up is recommended.

Genetic tests for methylene tetrahydrofolate reductase (MTHFR), plasminogen activator inhibitor type 1 (PAI-1), and 4G/5G PAI-1 promoter polymorphisms should not be ordered due to the null thrombotic risk associated with these conditions [18,48]. A summary of the recommended thrombophilia work-up and the limitations of these tests are depicted in Table 1.

Another important matter to address in these patients is the importance of ruling out cirrhosis, which would entail a context that requires a different management. Contrast-enhanced multiphase cross-sectional imaging studies (computed tomography scan [CT] or magnetic resonance imaging [MRI]), liver stiffness measurement, and/or liver biopsy are appropriate options for this purpose. The latter may not be necessary if the clinical suspicion of cirrhosis is high, especially if supported by pertinent risk factors, imaging showing liver segment IV atrophy, a clearly nodular surface, and/or a liver stiffness measurement >20 kilopascals. On the other hand, if there are no clinical or biochemical signs of cirrhosis, a liver biopsy may not be necessary unless there is a very specific reason for it, such as a concern for underlying PSVD [1,2,6].

Liver stiffness (LS) has been shown to be similar [49] or only slightly higher in patients with cavernous transformation of the portal vein after chronic PVT compared to age-matched healthy controls (median LS value of was 6.4 kPa (5.7–7.8) vs. 4.9 (4.0, 5.8), respectively), but significantly lower compared to patients with cirrhotic chronic PVT (17.7 [13.9, 30.8]) [50]. It can be measured by either transient or two-dimensional shear wave elastography [49,51].

MRI or CT scans can help differentiate cirrhosis from non-cirrhotic PVT by identifying classic imaging features of cirrhosis, which are generally not present in this condition. There may be some shared features between cirrhosis and late-stage chronic non-cirrhotic PVT, such as segment IV atrophy, caudate lobe hypertrophy, and peripheral atrophy, which has been hypothesized to be due to a reduction in the portal flow to the periphery with compensatory arterial flow, leading to a better perfusion of the central liver, and resulting in the described peripheral atrophy and central hypertrophy [52,53,54].

Ultrasound (US), CT scan, and MRI are the main imaging modalities currently used for establishing the diagnosis of PVT. US is a widely available, radiation-free, acceptable initial diagnostic modality when there is suspicion of PVT [55]. Although it is an operator-dependent study, a positive predictive value (PPV) of 86–97% and negative predictive value (NPV) of 98% have been reported [55,56]. In recent PVT, US would demonstrate heterogeneous material within the PV lumen and distension of the PV and tributary vessels, occasionally with an iso- or hypoechoic pattern. Doppler US would show partial or total absent flow in the PV lumen [57].

Contrast-enhanced CT scans and MRI are better and, thus, are recommended to define potential causes and complications of the PVT, as well as the extension of the thrombus into the portal venous system veins [55,57]. Regarding recent PVT, CT scan findings may include increased attenuation of the portal vein (PV) if no intravenous (IV) contrast is used; IV contrast would show a lack of PV enhancement after its administration. If an arterial phase is included, it would demonstrate hepatic enhancement in the arterial phase, with decreased hepatic enhancement during the portal phase. The PV may appear dilated with an acute, non-enhancing thrombus, with possible associated enhancement of the edge of the vessel because of local inflammation [57,58]. CT scans have a PPV of 99% and an NPV of 95% [56]. Multiphasic MRI offers the advantage of avoiding radiation while maintaining a comparable diagnostic accuracy to a contrast-enhanced CT scan, with a sensitivity of 100%, specificity of 99%, and accuracy of 99%, according to a study that included 36 patients with portal hypertension, most of them with cirrhosis [59]. PVT features include a filling defect with partial or complete occlusion in the PV lumen in the portal phase. Furthermore, MRI allows discrimination between bland and neoplastic PVT through diffused-weighted imaging [60]. T1-weighted imaging would demonstrate PVT isointensity to muscle, but occasionally it may show PVT hyperintensity in the acute phase. Similarly, in the acute phase, T2-weighted imaging would demonstrate a hyperintense signal [61]. 

**Table 1:** Thrombophilia investigations generally recommended after the diagnosis of non-cirrhotic PVT. Serum determination of antiphospholipid antibodies (anticardiolipin and anti-beta 2 glycoprotein antibodies), molecular testing, and CD55 and CD59 flow cytometry are not affected by acute thrombosis or anticoagulant treatment [6,13,62,63].

Thrombophilia	Considerations and Limitations
Myeloproliferative neoplasm	- Suspect if normal or elevated platelet count in the context of splenomegaly. Test for JAK2 V617F mutation. - If negative JAK2 V617F mutation but high clinical suspicion (platelets ≥450,000/uL, splenomegaly >15 cm), test for calreticulin gene mutation.
Antiphospholipid syndrome	- Diagnosis based on positive anticardiolipin antibodies, beta 2 glycoprotein antibodies, and/or lupus anticoagulant in the context of non-cirrhotic PVT, on two occasions separated by ≥12 weeks - Lupus anticoagulant, a series of coagulation screening tests, should never be performed in the acute context of a thrombotic event nor while the patient is on anticoagulant treatment.
Paroxysmal nocturnal hemoglobinuria	- Suspect especially if cytopenias, hemolytic anemia, and/or other unusual site venous thrombosis. - Testing consists of flow cytometry for glycosylphosphatidylinositol (GPI)-anchored protein deficiencies (CD55 and CD59) on the surface of blood cells.
Factor V Leiden	- Prothrombotic phenotype of the mutation is due to resultant activated protein C resistance. Molecular testing is confirmatory for factor V Leiden mutation.
Factor II G20210A mutation	- Prothrombotic phenotype of the mutation is due to resultant increased Factor II circulating levels. Molecular testing is confirmatory for Factor II G20210A mutation.
Protein C deficiency, Protein S deficiency, Antithrombin III deficiency	- Protein C, S, and anti-thrombin levels can be low in the setting of acute thrombosis, treatment with oral anticoagulants, or liver disease, without necessarily having an inherited deficiency. - Establishing a diagnosis of true deficiency requires normal baseline coagulation factor levels. Selection of the appropriate laboratory assay and interpretation of the results by a hematologist is highly recommended.

Collateral porto-portal vessels may become apparent in just a few weeks after an initial PVT event [13], but they are usually more evident on the chronic PVT spectrum. These venous collaterals may acquire a serpiginous appearance, which is known as cavernous transformation or portal cavernoma due to the radiologic appearance. In this scenario, the PV may not be well defined due to its replacement by collaterals [55,56,57]. Other possible accompanying features include linear calcifications within the thrombus, portosystemic collaterals, splenomegaly, and esophageal varices [57].

## 5. Natural History and Complications

In patients without cirrhosis, when a thrombotic occlusion of the PV trunk or its branches occurs, acute abdominal pain may or may not be present initially and, when it is, it is usually located in the upper abdominal region. The thrombotic occlusion can involve different extents of the PV system. If fever arises, it should raise suspicion for an acute intra-abdominal condition (diverticulitis, appendicitis, etc.). Ascites, usually subclinical or mild and transient, may also occur in the acute context [7,64], and it has been reported to take place more commonly in the context of concomitant Budd–Chiari syndrome [16,65]. Other potential presenting features include abdominal bloating, nausea, and splenomegaly [55], or those related to the underlying predisposing condition (inflammatory bowel disease, pancreatitis, etc.), if there was one. Although infrequently, esophagogastric varices (EVs) may occur as early as 1 month after the initial episode of PVT [64]. Liver function test abnormalities, if present, are usually mild and transient [7,13].

Extension of the thrombosis to the MV with mesenteric infarction should be suspected in the context of severe, ongoing abdominal pain, abdominal tenderness, and bloody stools, with associated organic failures (shock, acute kidney injury, metabolic acidosis, elevated lactate, mottled skin) [66]. However, recent PVT might be completely asymptomatic [13,67,68] and be found incidentally, although this is drastically more frequent in patients with cirrhosis (46% vs. 20%) and solid cancer (35% vs. 18%). Conversely, patients with unprovoked PVT (32% vs. 15%) or intra-abdominal inflammation/infection (14% vs. 6%) are significantly more likely to present with a clinical syndrome [69].

The current paradigm regarding the evolution of PVT sustains that after acute (recent) PVT, it may resolve spontaneously and full PV patency may be recovered, which, unlike in non-cirrhotic patients, happens in a high proportion of patients with cirrhosis (more commonly in non-occlusive PVT), and is directly related to the severity of liver disease [6,70,71]. Data regarding the percentage of patients with non-cirrhotic PVT that develop spontaneous recanalization are scarce, but it is considered uncommon [72].

In contrast to patients with cirrhosis, in patients with non-cirrhotic PVT, the increased resistance to portal flow is due to the obstructive thrombus, which can produce a significant opposition to portal flow even in the absence of a full occlusion of the PV lumen. According to Poiseuille’s law, a reduction of just 50% of the vessel lumen already leads to a marked increased pressure gradient [2].

When this is not accomplished, it can evolve into a chronic thrombus with development of porto-portal collaterals within a few weeks (which are very rare in patients with cirrhosis or PSVD) [2,13], receiving the name of cavernous transformation or cavernoma, which indicates that the PVT is not recent [13]. Portosystemic collaterals may develop as a consequence of portal hypertension, including rectal or ectopic varices, splenomegaly, and EVs [55,73], which represents the main morbidity associated with this condition. A classic cohort study by Turnes et al. including 36 patients reported that 64% developed EVs during follow-up, of which 55% were diagnosed after a median follow-up of 7 months (range, 1–40 months) [64]. Another study of patients with chronic non-cirrhotic PVT by Ferreira et al. revealed a 3-year estimated probability of developing EVs of 22%, and a 5-year risk of 32% of EV bleeding in patients with large EVs [73].

Although in the past some case-series studies have reported a hyperdynamic circulation (similar to cirrhosis) in non-cirrhotic PVT [74,75], currently it is uncertain whether these patients truly have objectively increased vasoactive systems and/or plasma volume expansion as would be expected in patients with cirrhosis-induced clinically significant portal hypertension, given that these clinical findings are not universal in these populations [2], which may respond, at least partially, to the widely recognized fact that the severity of the hyperdynamic circulation syndrome that occurs in patients with cirrhosis is directly proportional to the degree of liver dysfunction and consequent portal hypertension [76], an absent feature in patients with non-cirrhotic PVT. Furthermore, clinical ascites and hepatic encephalopathy are rare in this population, and when it happens it seems to be related to EV bleeding and/or portosystemic spontaneous shunts [77,78].

Portal cholangiopathy is a complication that may occur in patients with chronic PVT or portal cavernoma due to the porto-portal collaterals that these patients develop, which can compress the biliary system, causing ischemic bile duct injury, chronic inflammation, and fibrosis [79]. In general terms, it has been reported to be radiologically present in 77–100% of patients with chronic PVT [79], but only 5–20% of patients develop a clinical syndrome [80,81,82] (cholestatic manifestations ranging from elevated alkaline phosphatase to abdominal pain, hyperbilirubinemia, choledocholithiasis, and cholangitis), which occurs much more frequently in patients with a magnetic resonance cholangiography reporting biliary strictures with dilation [79]. Cholangiographic findings encompass shallow indentations, irregular ductal contours or angulations, upstream dilatation, ectasia, and bile duct angulations to strictures with frank dilatation [83]. It is considered a condition that presents late in the course of portal hypertension [80,81,82]; however, whether portal cholangiopathy by itself is a progressive condition or not in terms of liver fibrosis or biliary dysfunction is still a matter of debate [2,83].

Rarely, chronic or recurrent abdominal pain has been reported as a long-term complication in patients with chronic PVT. Considering the potential increased thrombotic risk in some of these patients, a contrast-enhanced CT scan should be considered to rule out recurrent PVT or extension to the MV [2,84] in the appropriate clinical context.

Finally, there are no studies examining the risk of hepatocellular carcinoma in this population, but it is considered to be low [13].

## 6. Treatment

In general, although evidence regarding specific scenarios is scarce, expert consensus recommends that if a predisposing factor or underlying thrombophilia is identified during diagnostic work-up, it should be treated accordingly. It must be acknowledged that, although biologically plausible, it remains largely unknown whether treating the underlying condition (when present) in fact decreases the risk of PVT recurrence for most of these conditions [2].

The Baveno VII consensus recommends that after the diagnosis of recent non-cirrhotic PVT, therapeutic-dose low-molecular-weight heparin should be started immediately as the primary treatment, with a switch to vitamin K antagonists (VKAs) when possible [1]. Non-fractioned heparin should be reserved for patients with diminished renal function, given the risk of heparin-induced thrombocytopenia reported in this population [1,85]. Direct oral anticoagulants (DOACs) are recommended as a primary option as well in the absence of triple positive APS, where they have been shown to be inferior to VKAs [86].

Recent studies suggest that direct oral anticoagulants (DOACs) are a safe and effective option for non-cirrhotic portal vein thrombosis (PVT), with comparable or superior outcomes to vitamin K antagonists (VKAs), despite the lack of direct comparative trials. However, the available evidence is based on heterogeneous patient populations, reflecting the diverse clinical scenarios in which PVT occurs. The underlying causes of PVT vary widely, including idiopathic cases, myeloproliferative neoplasms (MPNs), malignancies, intra-abdominal infections, recent surgery, inflammatory bowel disease, and estrogen therapy, among others.

For instance, an interventional cohort study evaluating rivaroxaban included a highly heterogeneous group (43% unprovoked, 28% with inflammatory or infectious conditions, 9% MPN, and 9% solid cancer), yet demonstrated a favorable safety profile with only 2% major bleeding and a high rate of thrombus improvement (83%) at three months [87]. Similarly, a retrospective study found DOACs to be associated with the highest complete radiologic resolution rates (65–75%) and a lower major bleeding risk compared to warfarin (HR 0.2), despite including patients with significant comorbidities such as intra-abdominal infections, JAK2-mutated MPNs, and inflammatory conditions [86]. A retrospective analysis by Ilcewicz et al. (2021) [88] further supports the safety of DOACs in patients with PVT. In a cohort of 33 patients with newly diagnosed PVT, including 10 with cirrhosis, 39.4% received DOACs, while the remaining patients received warfarin. No patients who received DOACs experienced a major bleeding event. However, the study lacked detailed information on PVT etiology in non-cirrhotic patients, limiting conclusions regarding specific risk profiles.

While VKAs remain widely used, large cohort studies reveal variability in outcomes depending on patient characteristics. The largest VKA-focused study in PVT found a major bleeding rate of 1.06 per 100 patient-years in unprovoked cases, though this risk could be higher in cirrhotic patients or those with additional thrombotic risk factors [89]. Similarly, an international registry study demonstrated a thrombotic recurrence rate of 6.3 per 100 patient-years in unprovoked PVT despite anticoagulation, reinforcing the need for individualized risk assessment [90].

A retrospective study by Kawata et al. [91] analyzed outcomes in both cirrhotic and non-cirrhotic PVT, including a sub-analysis of primary PVT (without cirrhosis). In this subgroup, DOACs demonstrated a similar efficacy and safety profile compared to VKAs, with no significant differences in thrombosis resolution or bleeding risk. However, the study’s small sample size and the broad inclusion of other PVT subtypes (including malignancy-associated and secondary PVT) limited direct comparisons, highlighting the need for more focused prospective studies in primary, non-cirrhotic PVT.

The most comprehensive assessment to date comes from a systematic review and meta-analysis by Li et al. [92], which included eight studies, three of which focused on non-cirrhotic patients [86,88,91]. In this subset, DOACs were associated with significantly lower major bleeding rates compared to no anticoagulation, low-molecular-weight heparin (LMWH), and VKAs, as well as higher rates of complete recanalization compared to VKAs. These findings strengthen the evidence base for DOAC use in non-cirrhotic PVT but also emphasize the need for randomized data to confirm these advantages in different PVT subtypes.

Although no randomized trials have directly compared DOACs and VKAs in PVT, current data suggest that DOACs offer at least comparable efficacy with potentially lower bleeding risks, particularly in non-cirrhotic patients. However, the heterogeneous nature of PVT and its underlying causes highlight the importance of individualized decision-making when selecting an anticoagulant strategy. Further prospective, comparative studies are needed to determine the optimal approach for different PVT subgroups, particularly in patients with MPN, malignancies, or those requiring long-term anticoagulation.

Evidence supporting this recommendation essentially comes from data showing that anticoagulant treatment is associated with higher rates of PV recanalization after recent PVT and decreased risk of MV extension, assuming that spontaneous resolution is unlikely to occur [7,72]. Nevertheless, according to those results, full-dose anticoagulant treatment may achieve recanalization in one-third to half of treated patients when given early in the disease course (<15 days after onset) [7,64,72]. Patients with ascites, occlusion affecting the PV trunk or both branches, and splenic vein occlusion are less likely to recanalize [7].

Anticoagulant treatment should be maintained for at least 6 months, and continued indefinitely in those with an underlying thrombophilia, but the Baveno VII consensus also endorses consideration for long-term treatment in those without it [1] in line with recommendations from other hematology/thrombosis experts, who recommend that the treatment approach should be guided by the whole clinical context (e.g., provoked vs. unprovoked PVT, unusual site thrombosis) rather than the presence or absence of an underlying thrombophilia [27,48,93]. A recent study by Baiges et al. including 114 patients with chronic non-cirrhotic PVT (48 with idiopathic/local risk factor-associated) demonstrated that patients without an identifiable thrombophilia, considered to have an isolated predisposing local risk factor, are indeed at high risk of splanchnic rethrombosis (25% incidence of rethrombosis) [94]. Similarly, a recent retrospective study by Ollivier-Hourmand et al. that included 154 patients with recent non-cirrhotic PVT associated with local risk factors found that long-term anticoagulation significantly reduces the incidence of new thrombotic events in patients with high-risk prothrombotic factors. At baseline, 53.9% of patients had at least one prothrombotic factor, with 32.5% having high-risk and 21.4% having low-risk factors. During follow-up, 45% of patients had at least one prothrombotic factor, with 33.6% being high-risk. Seventeen new thrombotic events occurred over a median follow-up of 52 months. High-risk prothrombotic factors were significantly associated with new thrombotic events (HR 3.817, 95% CI 1.303–11.180, *p* = 0.015). Permanent anticoagulation was found to be beneficial in preventing new thrombotic events. The HR for new thromboses was significantly reduced with anticoagulation (HR 0.976, 95% CI 0.956–0.995, *p* = 0.016). The authors conclude that the presence of high-risk factors requires long-term anticoagulation to prevent recurrent thrombosis [95].

It is worth reiterating that conditions such as MPN, PNH, and APS need to be ruled out because their presence would require specific treatment choices [93].

Regarding chronic PVT, anticoagulant treatment has been a matter of debate. Although historically it has been generally accepted that patients with an underlying thrombophilia require long-term anticoagulation, the necessity of anticoagulant treatment for those with chronic PVT or portal cavernoma without an identifiable permanent risk factor is less clear [96].

The benefit of long-term anticoagulation for the prevention of recurrent thrombosis in patients with unprovoked non-cirrhotic PVT was demonstrated by a prospective cohort study published in 2015, which showed that unprovoked splanchnic venous thrombosis was an independent predictor of recurrence [90]. These findings were further supported by a seminal randomized clinical trial published in 2022, where investigators included 111 patients with chronic non-cirrhotic PVT and found that in individuals without a “major risk factor” (defined as APS, MPN, homozygous Factor V Leiden, homozygous or composite heterozygous G20210A factor II, and a personal or first-degree family history of unprovoked venous thrombosis), rivaroxaban (15 mg/day) significantly reduced the recurrence of thromboembolic events (19/100 person-years vs. 0/100 person-years) without increasing the risk of major bleeding. Interestingly, they reported that D-dimer levels of <500 ng/mL measured 1 month after initiation of anticoagulant treatment were associated with a low risk of recurrent thromboembolism at 2 years [97].

Bleeding risk should always be accounted for and treatment individualized accordingly, but anticoagulant treatment has consistently shown to be safe in patients with non-cirrhotic PVT, where there may be concern for EV bleeding [90,97,98], and, moreover, it might even decrease overall mortality [98]. The Baveno VII consensus does specify that for patients with portal cavernoma, anticoagulation should be started after portal hypertensive bleeding prophylaxis has been established if high-risk varices are present [1].

Elevated Factor VIII level studies have shown mixed results over the last 3 decades regarding its role in thromboembolism recurrence prediction [99]. Factor VIII levels can be influenced by multiple circumstances, and cut-offs vary between populations, thus its value to predict the risk of recurrence is unknown [18]. Interestingly, the aforementioned observational study by Baiges et al. [94] showed an association between elevated Factor VIII (≥150%) and an increased risk of rethrombosis in a cohort of patients with chronic non-cirrhotic PVT.

These assessments may assist the clinician with the decision of the appropriate dosing and duration of anticoagulant treatment for these patients [1,3,94]. With the mentioned nuances, there will probably be several different appropriate regimens for individual scenarios.

### 6.1. Portal Hypertension

As mentioned in the Natural History Section, patients with non-cirrhotic PVT can develop EVs as soon as 1 month after the initial event [64]. Thus, it is recommended that every patient with recent PVT undergoes upper endoscopy. A follow-up CT scan performed 6 months after recent PVT is recommended [1]. If recanalization is not achieved by then, patients should be screened again for EVs. In the absence of varices, they recommend repeating upper endoscopy at 12 months and at 2 years afterward. In chronic non-cirrhotic PVT, the Baveno VII consensus suggests following recommendations for cirrhotic patients in case of acute variceal bleeding and regarding the use of non-selective beta blockers and endoscopic band ligation for secondary prophylaxis of variceal bleeding in these patients [1]. Interestingly, a relatively recent study found that treatment with VKAs does not increase bleeding risk significantly during endoscopic band ligation in patients with chronic non-cirrhotic PVT and, thus, the Baveno VII consensus suggests that EV band ligation can be safely performed without stopping VKAs prior to these procedures in this group [100]. Finally, in cirrhotic patients, carvedilol has been shown to decrease portal pressure significantly more than propranolol. This is considered to be due to the additional unique alpha-1 blocker effect this medication possesses, which decreases vascular hepatic resistance, the latter being normal (or not significantly increased) in patients with non-cirrhotic PVT. Thus, although not analyzed in patients with non-cirrhotic PVT, propranolol may be equally effective compared to carvedilol outside the setting of cirrhosis [2,90]. Transjugular intrahepatic portosystemic shunts (TIPSs) with or without PV recanalization have been increasingly used in these patients with very good results for decreasing portal hypertension-related complications, especially in patients with endoscopic-refractory variceal bleeding [84,101].

Interventional radiology procedures play a significant role in two settings: when anticoagulation alone is not sufficient (or contraindicated) or when there is imminent risk of intestinal ischemia; therapeutic options typically include TIPS placement and/or local thrombolysis (with or without thrombus fragmentation). Under the assumption that early anticoagulation for recent non-cirrhotic PVT may achieve recanalization in up to ~50% of treated patients [7,62,72], and given the fact that the MPN-associated non-cirrhotic PVT subgroup has an even smaller rate of recanalization with anticoagulant treatment [102], and that the absence of recanalization is associated with chronic PVT complications (portal hypertension, portal cholangiopathy, MV extension, and abdominal pain), interventional radiology PV recanalization has been increasingly used and has been shown to be safe and effective in experienced centers [101,102,103].

A recently published retrospective study reported the experience of a single US center using TIPS combined with thrombectomy for the treatment of recent non-cirrhotic PVT [103]. The median age was 51 years (range 39–62 years), and patients were followed for 1 year. The causes of PVT were idiopathic (*n* = 12), prothrombotic disorders (*n* = 11), postsurgical sequelae (*n* = 6), pancreatitis (*n* = 2), and Budd–Chiari syndrome (*n* = 1), and the indications for TIPS-thrombectomy included refractory abdominal pain (*n* = 14), intestinal venous ischemia (*n* = 9), ascites (*n* = 4), high-risk varices (*n* = 3), and variceal bleeding (*n* = 2). They achieved a 100% success rate for recanalization of the portal vein, with three procedure-related adverse events (Society of Interventional Radiology grade 2 moderate), and one patient developed hepatic encephalopathy post-TIPS placement.

The 1-year primary and secondary patency rates for the portal vein and TIPS were 78% and 100%, respectively, and three (9%) patients experienced a return of symptoms, including ascites, variceal bleeding, and intestinal venous ischemia at a 1-year follow-up.

Similarly, a Chinese study including 25 patients with acute severe NC-PMVT treated with TIPS in conjunction with mechanical thrombectomy and dual-access thrombolysis recently reported technical success in 100% of the patients (40% fully recanalized, 60% partially recanalized) [104]. No significant procedure-related complications were reported. Both studies concluded that TIPS-thrombectomy is safe and effective for patients with acute severe NC-PMVT, offering high recanalization rates and manageable complication profiles. Notably, both studies used anticoagulation after the procedures, without specifying duration of the treatment.

In cases of chronic non-cirrhotic PVT, a TIPS is an important intervention for managing refractory complications of portal hypertension despite adequate anticoagulation. Additionally, though less frequently, it is utilized for the alleviation of intractable abdominal pain, particularly when the thrombus extends to the superior mesenteric vein. A systematic review and meta-analysis by Rodrigues et al. [105] demonstrated that a TIPS is highly feasible, effective, and safe for portal vein recanalization in patients with chronic PVT (technical success rate of TIPSs in these patients was approximately 95%, with a 12-month portal vein recanalization rate of 79% and a shunt patency rate of 84%), although only 8% were non-cirrhotic patients. Many other retrospective studies in non-cirrhotic patients with chronic PVT have demonstrated that a TIPS is a feasible and effective treatment for managing complications of portal hypertension, including those with portal cavernoma. The procedure is linked to high technical success rates and notable clinical improvements. However, maintaining shunt patency presents a challenge that may necessitate additional interventions, usually in young patients with preserved hepatocellular function [84,106]. Table 2 summarizes the initial diagnostic and therapeutic modalities for these patients.

### 6.2. Portal Cholangiopathy

Treatment is limited to (and guided by) signs or symptoms resulting from this condition, such as cholangitis, choledocholithiasis, pancreatitis, jaundice, and pruritus. It should include a multidisciplinary team discussion at experienced centers. Options include ursodeoxycholic acid, endoscopic biliary drainage, interventional radiology-performed portal vein recanalization, and portosystemic shunt surgery if shuntable veins are present. In general, it is aimed at relieving the biliary obstruction, which may benefit from concomitant portal vein recanalization in some settings, a field where interventional radiology-performed portal vein recanalization has recently shown safe and effective results, but the whole clinical picture as well as the center experience must be considered to define the best individual approach [2,3,81,84,107].

## 7. Conclusions

PVT in non-cirrhotic individuals entails a different pathophysiology, and thus, diagnostic approaches and treatment strategies, compared to patients with cirrhosis. It presents a unique clinical challenge given its distinct pathophysiology and the diverse range of underlying risk factors. Recent advancements in our understanding have highlighted the importance of early diagnosis and tailored treatment strategies to improve patient outcomes. Anticoagulant therapy remains a cornerstone in the management of recent PVT, with evidence supporting its role in achieving portal vein recanalization and preventing mesenteric vein extension. However, the management of chronic PVT and unprovoked case requires a more nuanced approach, with long-term anticoagulation being considered even in the absence of clear provoking factors or underlying thrombophilia.

Future research should focus on identifying specific patient populations that would benefit most from long-term anticoagulation and developing strategies to manage those who do not achieve portal vein patency with anticoagulation. Additionally, the role of interventional radiology in the recanalization of the portal vein and the management of portal hypertension-related complications warrants further exploration to better define its role and timing.

## Figures and Tables

**Table 2:** Summary of initial diagnostic and therapeutic approach for patients with non-cirrhotic PVT.

	Diagnostic Modalities
Doppler Ultrasound	- Appropriate for initial suspicion - Operator-dependent results - Radiation-free, widely available, non-expensive option
CT scan and MRI	- Widely available - Excellent to define extension and/or local associated complications of PVT - Useful for interventional therapy planning
	**Therapeutic Modalities**
Anticoagulant Therapy	- Recent PVT: LMWH (more data available) or DOAC for initial treatment (except in APS). VKA and DOAC (except in APS) after initial LMWH are acceptable choices. Length of therapy should be at least 6 months. A total of 30–50% achieve recanalization. - Consider long-term anticoagulant treatment for all patients at high risk of recurrent PVT. Evidence is more conclusive in patients with recent PVT, but chronic PVT may also benefit from this strategy [97]. An individualized approach considering thrombotic and bleeding risk is mandatory. - Factor VIII (≥150%) and D-dimer (<500 ng/mL) levels may aid in the decision to withhold anticoagulation after 6 months when in doubt; more research needed to define their exact role.
Portal Hypertension Management	- **Recent PVT**: Perform upper endoscopy for all patients. A follow-up CT scan should be performed at 6 months. If recanalization not achieved by then, screen again for EVs. In the absence of varices, repeating upper endoscopy at 12 months and at 2 years afterwards is recommended. - **Chronic PVT:** management should follow guidelines for cirrhotic patients, including acute variceal bleeding treatment, use of non-selective beta blockers (unclear if carvedilol is superior to propranolol in non-cirrhotic patients), and endoscopic band ligation for secondary prophylaxis of variceal bleeding.
Interventional Management	- **TIPS**: To decrease portal hypertension-related complications, especially in patients with endoscopic-refractory variceal bleeding; very good results in experienced centers. Combination of TIPS and thrombectomy has been used for recent PVT with refractory abdominal pain, intestinal venous ischemia, ascites, high-risk varices, and variceal bleeding with excellent results [103]. - **Local thrombolysis (with or without thrombus fragmentation)**: particularly useful when there is high risk of intestinal ischemia.
